# Social inequality in health: revisiting moments and trends in 50 years of publication of RSP

**DOI:** 10.1590/S1518-8787.2017051000156

**Published:** 2017-02-21

**Authors:** Marilisa Berti de Azevedo Barros

**Affiliations:** I Departamento de Saúde Coletiva. Faculdade de Ciências Médicas. Universidade Estadual de Campinas. Campinas, SP, Brasil

**Keywords:** Health Inequalities, Publications, trends, Public Health, Review, Historical Article

## Abstract

This study describes the frequency and types of articles on social inequalities in health published in 50 years of the *Revista de Saúde Pública*, taking as reference some milestones that were used as guidelines to develop the research on this theme. Checking titles, keywords and abstracts or full texts, we identified 288 articles whose central or secondary focus was social inequalities in health. Corresponding to just 1.8% in the initial years, articles on social inequalities in health have represent 10.1% of the articles published in the last decade. The designs used were mainly cross-sectional (58.0%) and ecological (18.1%). The most analyzed themes were: food/nutrition (20.8%), mortality (13.5%), infectious diseases (10.1%), oral health (9.0%), and health services (8.7%). Articles focused on the analysis of racial inequalities in health amounted to 6.9%. Few articles monitored the trends of social inequalities in health, essential enterprise to assess and support interventions, and an even smaller number evaluated the impact of policies and programs on the reduction of social inequalities in health.

## RESUMO

Este estudo descreve a frequência e os tipos de artigos sobre desigualdades sociais em saúde publicados nos 50 anos da Revista de Saúde Pública, tomando por referência alguns marcos que balizaram o desenvolvimento das investigações nessa temática. Checando títulos, palavras-chave e resumos ou textos completos, foram identificados 288 artigos cujo foco central ou secundário era desigualdades sociais em saúde. Correspondendo a apenas 1,8% nos anos iniciais, artigos sobre desigualdades sociais em saúde chegaram a representar 10,1% dos publicados na última década. Os desenhos utilizados foram principalmente transversais (58,0%) e ecológicos espaciais (18,1%). Os temas mais analisados foram: alimentação/nutrição (20,8%), mortalidade (13,5%), doenças infecciosas (10,1%), saúde bucal (9,0%) e serviços de saúde (8,7%). Artigos voltados à análise de desigualdades raciais em saúde somaram 6,9%. Poucos artigos monitoraram as tendências das desigualdades sociais em saúde, empreendimento essencial para avaliar e subsidiar intervenções, e um número ainda menor avaliou o impacto de políticas e programas na redução das desigualdades sociais em saúde.

## INTRODUCTION

Observations on the influence of living conditions in the health of populations have been recorded since antiquity, but it is in the 19th century that data on this issue gain consistency and increase with the social medicine movement and the work of the social reformers^28^. From global approaches, which prevailed in the middle of the 19th century with the aim of understanding the causes of diseases and epidemics, the emergence of the bacteriological era led the focus of research to the identification of pathogens and to the ways of prevention and control. Investigations with greater social scope, including the living conditions of the population, gain importance again after World War II, when cardiovascular diseases, neoplasms, accidents and violence became priorities in the morbimortality setting^34^. The understanding of the causes of these problems requires to take into account the people’s material conditions and lifestyle, although this widening of perspective does not imply necessarily in identifying the differences ascertained as being socially determined^17^.

The issue of social inequalities in health (SIH) gained relevance in the course of the 20th century, by finding deep inequalities in living conditions and by the consistent results of researches that measured the social disparities in patterns of health-disease and access to health services^35^. Data also showed that these inequalities cross the entire social fabric, not restricted to the segment situated below the poverty line^10^.

In global terms, the Declaration of Alma Ata, in 1978, already highlighted the importance of interventions to promote greater equity in the access to health services, emphasizing, in this perspective, the role of social and health policies. Another milestone, within the theme of SIH, is the wide impact of the findings and controversies generated by the report of the Working Group on Inequalities in Health coordinated by Sir Douglas Black, in England, in 1980^35^. The report brought to the debate the boundaries of public health services in achieving equity in health, the need of monitoring the SIH and the importance of accurate measurement of these disparities.

The need to identify social strata and the difficulties and limitations inherent in this journey are present in the first articles about SIH published by *Revista de Saúde Pública* (RSP). Only 1.8% of the articles from RSP in its first decade focus on the issue of social inequalities in health (Table 1), and many of them develop theoretical-conceptual discussion or present proposals for social stratification^2^ ^,^ ^38^.

**Table 1:** Articles on social inequalities in health, published in *Revista de Saúde Pública* according to the decades of the journal’s existence.

Period	Fascicles	Special issues or supplements	Total	Articles published in RSP	Articles about SIH	
			
	n	n		n	n	%
1967-1976	30	3	33	341	6	1.8
1977-1986	52	5	57	503	17	3.4
1987-1996	60	2	62	649	36	5.6
1997-2006	60	6	66	1.128	96	8.5
2007-2016	60	11	71	1,312	133	10.1

Total	262	27	289	3,933	288	7.3

RSP: *Revista de Saúde Pública*; SIH: social inequalities in health

In Brazil and in Latin America, movements in the field of health, aligned with broader social movements that acted against the military dictatorships spread in the region during the 1960s and 1970s, brought new perspectives for understanding health profiles and the organization and role of health services. These movements built the field of Collective Health in Brazil, which, in September 1979, result in the foundation of the Brazilian Association of Collective Health (ABRASCO)^19^ ^,^ ^29^. The Latin American production in this period, fueled by historical materialism concepts, privileges, in relation to the production and distribution of health and disease, the analyses of health inequalities among social classes and the consequences of work conditions and social work relationships in the health of workers^11^ ^,^ ^18^.

Following this approach, some articles published in RSP show proposals and applications of the operationalization of the Marxist concept of social class, with the aim of analyzing SIH among significant segments of the capitalist societies instead of among strata arbitrarily chosen^6^ ^,^ ^20^. These studies were aligned with the proposals of Critical Epidemiology and Social Epidemiology^11^ ^,^ ^18^ and several articles using social class were published in RSP.

The development of studies on SIH depends, in addition to the motivation of researchers and the support of funding agencies, on the existence of valid and complete information of social variables in available databases. The absence of these conditions limited the development of studies on this subject, which is consistent with the small number of articles on SIH published in the early decades of RSP (Table 1). In 1996, the World Health Organization launched an initiative to face health inequities considering the existence of unacceptably large social disparities in health, still increasing in several countries, and the expenditure constraint with social policies. An issue highlighted in the document is precisely the lack of essential information in the usual databases of the countries that could facilitate the monitoring of the SIH^39^.

Worldwide, the production of studies on SIH has grown substantially since the 1990s, duplicating the number of publications every four years^9^. Also in RSP, the percentage of articles focusing on SIH rose from 1.8% in the first decade to 10.0% in the last (Table 1). Several studies included in the category of SIH did not have as central focus the analysis of social disparities or of equity in health. As it is almost inevitable to use social and economic variables in analyzing health themes, and that these variables exert strong determination in health, many articles – whose main purpose was not to analyze SIH - ended up emphasizing among their results and in the abstract, findings of this nature and thus they were included in the roll of articles analyzed. The procedures adopted for the selection of articles are described in the Figure.

**Figure:**
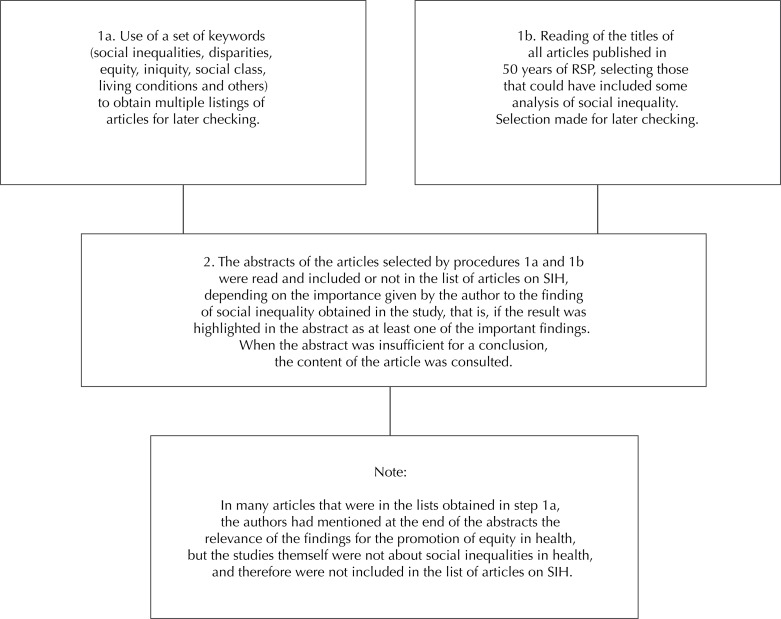
Procedures for the selection of articles on Social Inequalities in Health of *Revista de Saúde Pública*.

RSP: *Revista de Saúde Pública*; SIH: social inequalities in health

More recently, the World Health Organization’s initiative to stimulate the creation of Commissions on Social Determinants of Health contributed to the renewed emphasis on research about SIH. The proposal assumed that the development of a society should be judged by the quality of health of its population, by the way which health is distributed among the social strata, and by the degree of protection provided to the socially disadvantaged sectors. The initiative, implemented in many countries, culminated in the holding of a Global Conference in Rio de Janeiro in 2011. The work of the commissions allowed resumption of the discussion on theories, concepts, methods and, in particular, on the need for the issue of inequity in health to be effectively incorporated in the political agendas of the governments^13^ ^,^ ^22^.

The increase in the number of articles published and the expansion of the themes investigated from the perspective of SIH in 50 years of RSP accompany the advance of the qualification of existent databases^24^, the strengthening of post-graduation programs in Public Health and consolidation of research groups^5^ ^,^ ^15^, as well as the implementation of local and national surveys^7^. The themes analyzed in the last decade cover almost all objects of health, including mortality, communicable and non-communicable diseases, health limitations and disabilities, oral and mental health, self-evaluated health, health-related behaviors, and the access and use of different types of health services (Table 2). With high and persisted frequency throughout the period, we found studies of social inequalities in feeding and nutrition, which are 20.8% of the total. The relevance of nutritional deficiencies at the beginning of the period, of obesity in recent decades, and the recognition of the importance of feeding profile on the incidence of non-communicable chronic diseases explain the preponderance of this subject in relation to the others. This preponderance is also justified by the existence of consolidated research groups and by the availability of data from national surveys, such as the Household Budget Survey (HBS), among others.

**Table 2:** Articles on social inequalities in health, published by *Revista de Saúde Pública* according to theme and the decades of the journal’s existence.

Object/Theme	1967-1976		1977-1986		1987-1996		1997-2006		2007-2016		Total	
					
	n	%	n	%	n	%	n	%	n	%	n	%
Nutrition/Food	1	16.6	9	52.9	13	36.1	16	16.7	21	15.8	60	20.8
Mortality	-	-	2	11.8	7	19.4	15	15.6	15	11.3	39	13.5
Infectious and parasitic diseases	-	-	-	-	4	11.1	9	9.4	16	12.0	29	10.1
Oral Health Care	2	33.3	-	-	-	-	6	6.3	18	13.5	26	9.0
Health services	-	-	-	-	1	2.8	10	10.4	14	10.5	25	8.7
Chronic diseases and Behaviors	-	-	-	-	2	5.5	9	9.3	12	9.0	23	8.0
Health conditions	-	-	1	5.9	2	5.5	2	2.1	8	6.0	13	4.5
Mental Health/Violence/Accidents	-	-	-	-	-	-	6	6.3	5	3.7	11	3.8
Hospitalization	-	-	-	-	1	2.8	7	7.3	2	1.5	10	3.5
Medication	-	-	-	-	-	-	3	3.1	4	3.0	7	2.4
Conceptual/Methodological	3	50.0	4	23.5	6	16.7	4	4.2	8	6.0	25	8.7
Others	-	-	1	5.9	-	-	9	9.3	10	7.5	20	6.9

Total	6	100	17	100	36	100	96	100	133	100	288	100

In addition to mortality (13.5% of articles), which first article was published in 1980^26^, and infectious diseases (10.1%), whose databases are available, another theme with strong presence in the RSP is oral health, which included also articles on the access and use of oral health services (Table 2). The great frequency of articles on this subject is due to the intense social inequality in the access to these services that still prevails in the Country, the conduct of national surveys, such as the 2010 National Oral Health Survey and the one held in 2002/2003, and the existence of consolidated research groups. By the way, the first issue of the RSP has an article on the different prevalence of dental caries among white and non-white schoolers^33^.

Considering the study designs, the majority (58.0%) consists of cross-sectional studies and mainly from household surveys. Other frequent design is the ecological studies that analyze health conditions of populations territorially referenced and comprise 18.1% of the articles on SIH (Table 3). In these approaches, the authors assume the space not only in its natural or ecological administrative dimension, but also as social and historically constituted^25^. The spatial units of analysis adopted were the most diverse, mainly municipalities (21.1%) and areas within the cities (57.7%). The themes analyzed were mortality (44.2%) and infectious diseases (32.7%) and, among them, HIV and dengue. In addition to education and income, several compound indexes were used in these articles, including: human development index (HDI), condition of life, Sao Paulo indexes of vulnerability and of social responsibility, unmet basic needs, Gini index, Theil inequality, urban quality, among others.

**Table 3:** Ecological articles on social-spatial inequalities in health, according to the period of publication of *Revista de Saúde Pública.*

Period	Articles about SIH	Spatial ecological	

		n	%
1967-1976	6	0	-
1977-1986	17	1	5.9
1987-1996	36	7	19.4
1997-2006	96	16	16.7
2007-2016	133	28	21.1

Total	288	52	18.1

SIH: social inequalities in health

The evaluation of the effect of the degree of income inequalities on health, regardless of per capita income, went on to establish a research field with strong development in Europe and United States^37^, but with little expression on the production published in RSP. However, more recent articles, ecological or with multilevel analysis, incorporated variables or indexes of inequality income distribution^12^ ^,^ ^16^.

In studies of individual based approach, the socioeconomic variables used for definition of strata or categories of comparison were mainly income and education. Some articles also add housing conditions, number of equipments, and very few used occupational categories or groups. The majority used social stratification for their analysis of inequality instead of social class approaches^3^ ^,^ ^4^.

In addition to the health inequities related to socioeconomic strata, the literature has highlighted the increasing focus on inequalities concerning race, ethnicity and gender, among others^8^ ^,^ ^17^. Of the articles about SIH published in RSP, 14.9% (43) analyzed variables of color/race/ethnicity and 6.9% (20) had this inequality as their central focus (Table 4). On this theme, a more recent and conceptual article was published in RSP^27^. The number of articles focusing on racial or social discrimination and gender inequalities is rather small. The scarce production of studies on racial, ethnic, and gender inequalities in health was also observed in a bibliometric article analyzing the production in SIH in Latin America and Caribbean^1^.

**Table 4:** Articles on racial inequalities in health according to the period of publication of *Revista de Saúde Pública.*

Period	Articles about SIH	Articles that analyze race/color/ethnicity (all)		Articles focusing on inequalities by race/color	
	
		n	%	n	%
1967-1976	6	2	33.3	2	33.3
1977-1986	17	1	5.8	1	5.8
1987-1996	36	2	5.5	2	5.5
1997-2006	96	8	8.3	5	5.2
2007-2016	133	30	22.5	10	7.5

Total	288	43	14.9	20	6.9

SIH: social inequalities in health

In Brazil, the existence of the Unified Health System (SUS) and the possibility of comparing groups according to the funding sources of the health services or by the public or private nature of the health establishments, represented strategies used by authors to scale the degree of health disparities. These analyses allowed us to infer about the potential and the effectiveness of the services provided by SUS in reducing health inequality. Nineteen articles of SIH used this strategy of analysis and some pioneers applied this approach even before the foundation of SUS, comparing patients according to funding source^36^ and reminding the existence of the category “destitute” which was used in that period^32^.

Developed countries are considered to have produced sufficient information about the magnitude of the social inequalities in health, and the researches in the area moved from description to search for explanation and, now, are leading to the evaluation of the effectiveness of interventions and policies aimed at reducing the iniquities in health^21^ ^,^ ^30^. However, despite the large amount of published articles about SIH and the wide subject covered, we do not have a clear and consistent picture about the magnitude of the SIH in different indicators and contexts in the Brazilian reality, lack that some initiatives tried to overcome^13^ ^,^ ^14^. More importantly, SIH trends were analyzed in only 19 articles that showed stability, increase or decline, depending on the indicator analyzed^31^. The monitoring of SIH is essential to detect moments and circumstances of the changes of health inequalities, providing clues to the identification of possible determinants. Few were the articles that evaluated the impact of programs and policies in SIH^23^.

In synthesis, we observed the increase of published articles about SIH, the advancement and increased complexity of methods and analytical techniques used, and the expansion of the themes explored. It is clear, however, the need for productions that generate a more systematized knowledge on health inequities in Brazil. We also observed a lack of conceptual and theoretical studies, of trend analysis and monitoring of SIH and of investigations about the effect of policies and interventions aimed to reduce social differences in health.
